# Supplementary figures and images for: HL-IR mediates cinnamaldehyde repellency behavior in parthenogenetic Haemaphysalis longicornis
Source: PLoS Negl Trop Dis. 2025 Mar 17;19(3):e0012877. doi: 10.1371/journal.pntd.0012877 (PMC11913321; doi:10.1371/journal.pntd.0012877)

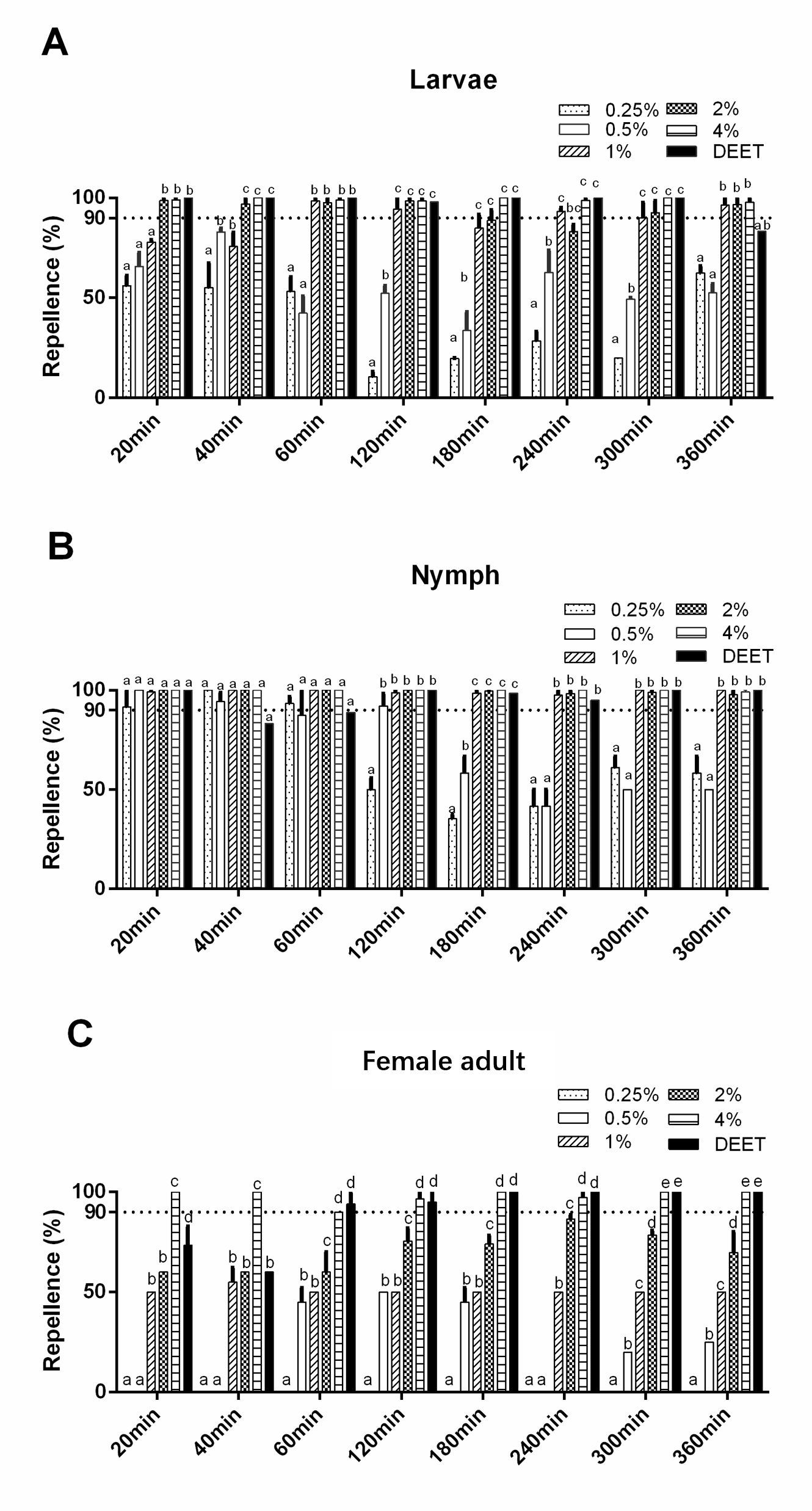

Supplement: S1 Fig — Data were analyzed by ANOVA, Dunnett’s Multiple Comparison-test, and differences are indicated by different letters. A. Cinnamaldehyde repellency test within 6 hours in parthenogenesis larvae. The mean repellency of cinnamaldehyde at concentrations of 0.25%, 0.5%, 1%, 2%, and 4% over a 360min period was 39.12%, 54.90%, 87.56%, 94.03%, and 99.14%, respectively. Notably, the 4% concentration of cinnamaldehyde achieved over 90% repellency at all time points within the 360min duration. In the positive control group, the mean repellency of 20% DEET for the same period was 97.67%. B. Cinnamaldehyde repellency test within 6 hours in parthenogenesis nymph. The average repellency of cinnamaldehyde at the 0.25%, 0.5%, 1%, 2%, and 4% concentrations was recorded as 68.22%, 76.06%, 99.36%, 99.32%, and 95.72%, respectively. The 1%, 2%, and 4% concentrations of cinnamaldehyde maintained repellency rates exceeding 90% at all time points during the 360min assay. The mean repellency of 20% DEET during this stage in the positive control group was 95.72%. C. Cinnamaldehyde repellency test within 6 hours in female adult. The average repellency rates of cinnamaldehyde at concentrations of 0.25%, 0.5%, 1%, 2%, and 4% over the 6-hour period were 0%, 21.76%, 50.59%, 69.91%, and 98.16%, respectively. The 4% concentration of cinnamaldehyde consistently achieved over 90% repellency at all time points, indicating a significant repellent effect on female adult. The average repellency rate of 20% DEET for 6 hours in the positive control group was 91.84%. (TIF) [file pntd.0012877.s002.tif]

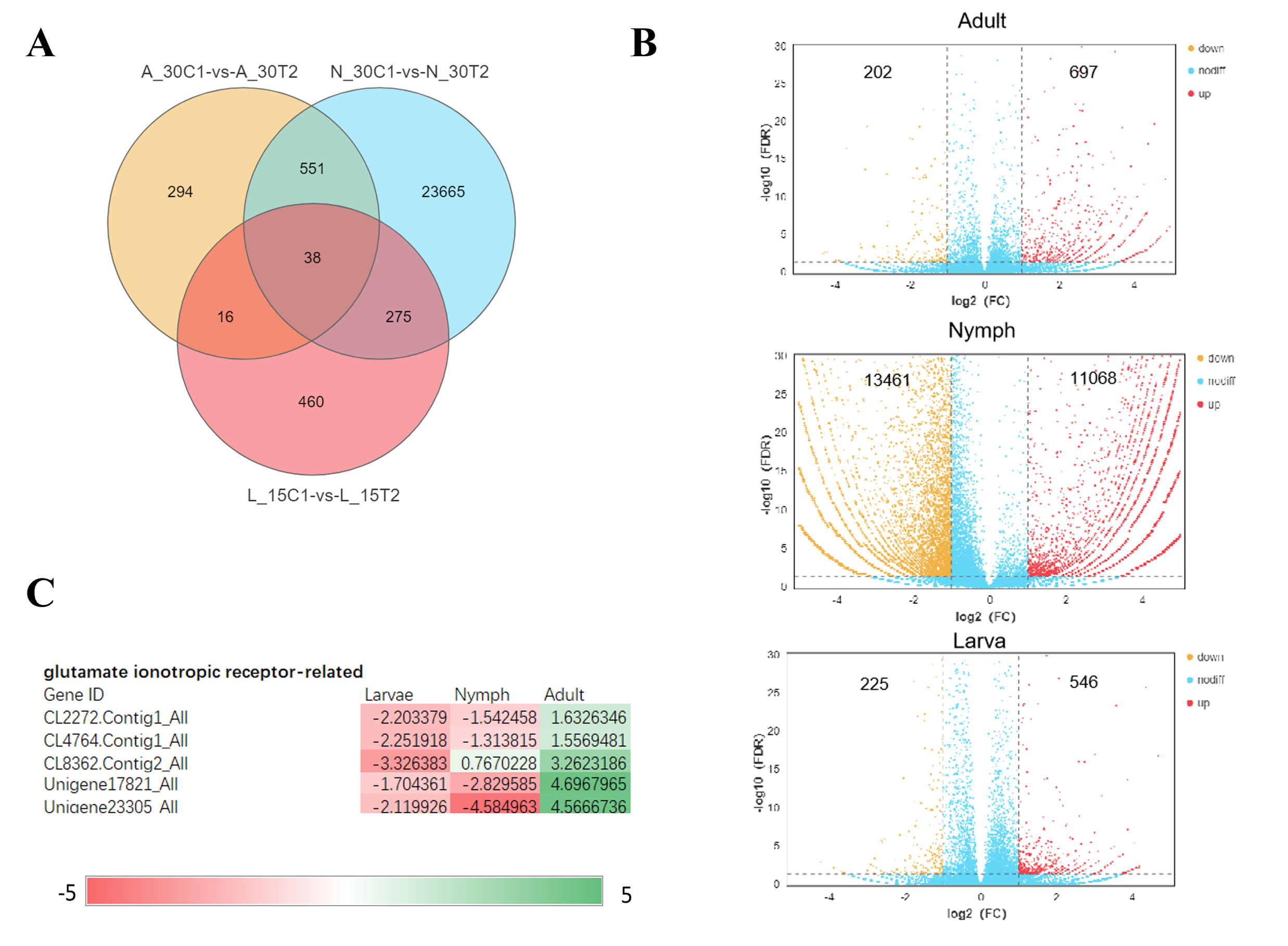

Supplement: S2 Fig — A. Venn analysis of the number of differential transcripts of H. longicornis at different developmental stages. A_: female adult, N_: nymph, L_: larvae, C1: control group, T2: treat group. B. Number of differentially expressed genes between unstimulated and stimulated ticks in different developmental stages. Red (upregulated) and yellow (downregulated). Adult: female adult, Nymph: nymph, Larvae: larvae. C. Cinnamaldehyde stimulates changes in transcript levels of IR-related genes at different developmental stages. The bar indicates (l-r) downregulated (red) to upregulated (green) with −5 <log2 normalized fold change < 5. (TIF) [file pntd.0012877.s003.tif]

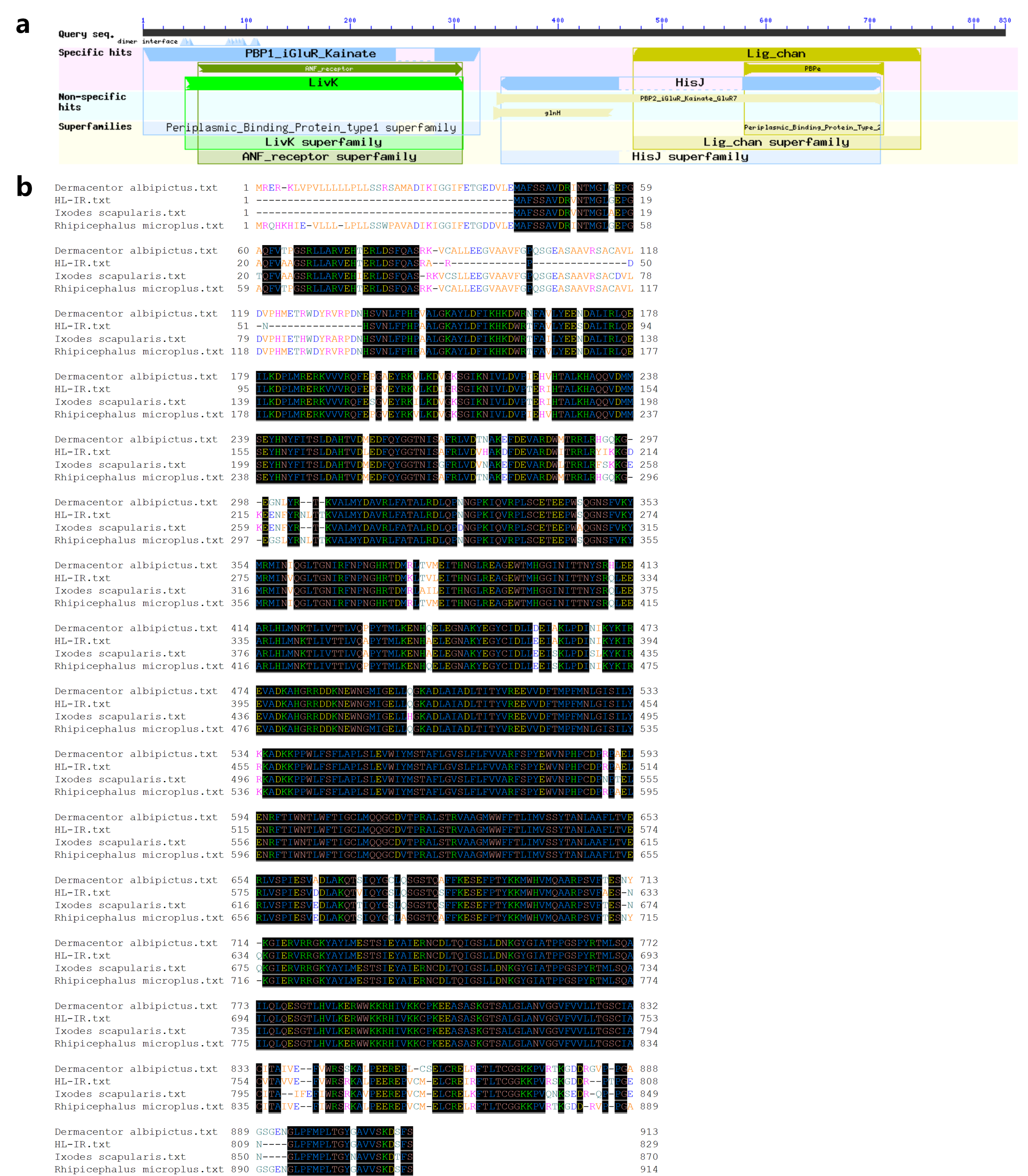

Supplement: S3 Fig — A. Conserved domain analysis results for HL-IR. B. Alignment of HL-IR amino acid sequences with those of other species. (TIF) [file pntd.0012877.s004.tif]

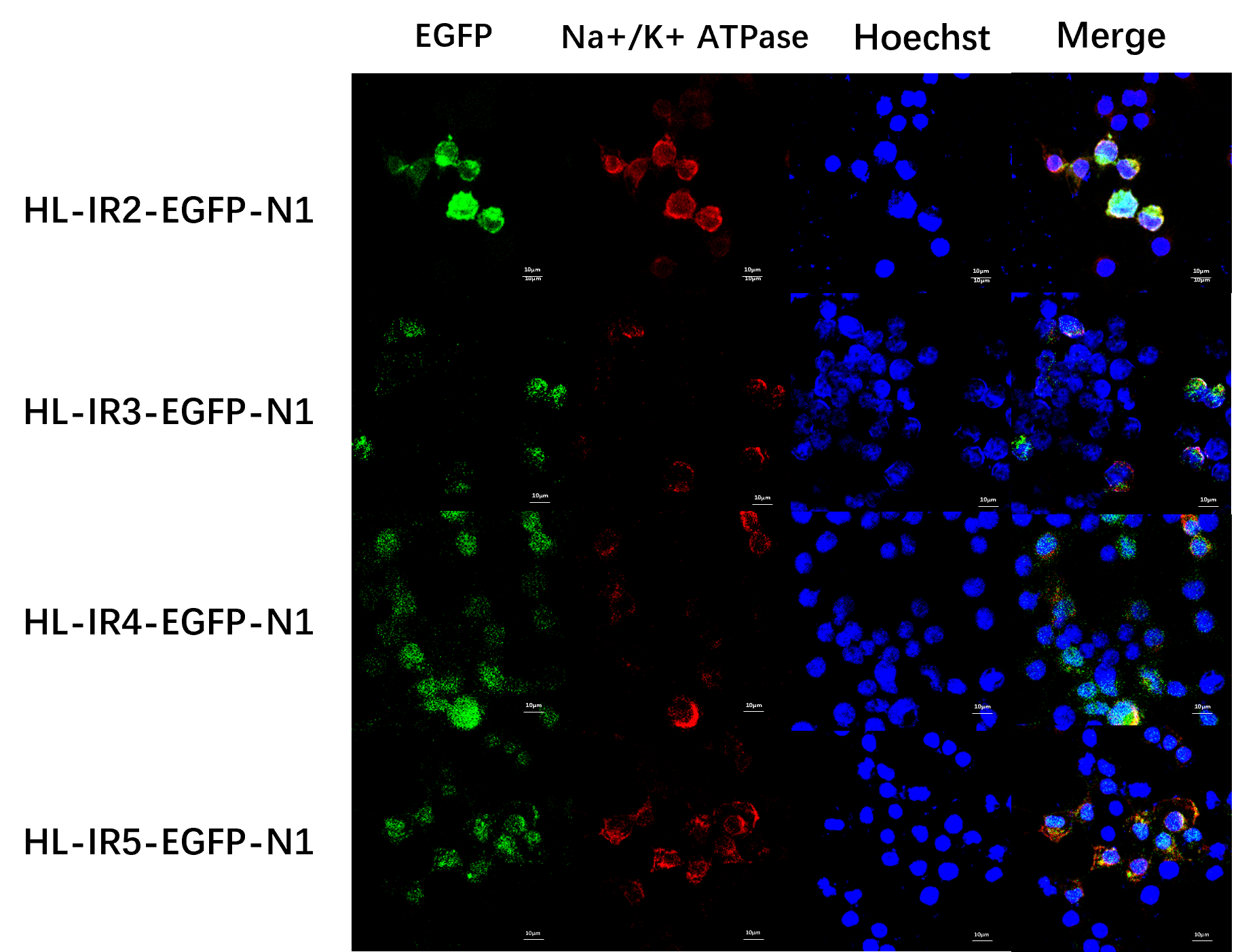

Supplement: S4 Fig — Green Fluorescence: the protein containing the EGFP fluorescent tag. Red Fluorescence: Na+/K+-ATPase (Cell membrane). Blue Fluorescence: Cell nucleus. The scale bars = 10 μm. (TIF) [file pntd.0012877.s005.tif]
