# Supplementary material for: HL-IR mediates cinnamaldehyde repellency behavior in parthenogenetic Haemaphysalis longicornis
Source: PLoS Negl Trop Dis. 2025 Mar 17;19(3):e0012877. doi: 10.1371/journal.pntd.0012877 (PMC11913321; doi:10.1371/journal.pntd.0012877)
Supplement: Attachment — Submitted filename: Responses to the comment.docx [file pntd.0012877.s007.docx]

**RESPONSE TO THE COMMENTS**

**Summary and General Comments**


Use this section to provide overall comments, discuss strengths/weaknesses of the study, novelty, significance, general execution and scholarship. You may also include additional comments for the author, including concerns about dual publication, research ethics, or publication ethics. If requesting major revision, please articulate the new experiments that are needed.

Reviewer #1: (No Response)

Reviewer #2: This study analyzed the repellent effect of cinnamaldehyde in Haemaphysalis longicornis and its correlation with HL-IR. It is a well-designed experiment and the results are interesting. However, for a clearer analysis of the results, this reviewer suggests the following modifications.

1. The Introduction is missing a lot of information about the purpose and background of the experiment. For example, there is no explanation of why cinnamaldehyde, the chemical used in the test, was chosen. There is only a partial description of Chinese cinnamon oil.
**Re:** **In previous study, we did a compositional analysis of cinnamon essential oil and found that the main component is cinnamaldehyde. We add this information at L62.**

2. The origin of the H. longicornis used in this study should be explained.
**Re: We added the origin of the *H. longicornis* in L86.**

3. Many ticks were used in this experiment, and the batch information of the ticks is missing. Are all the ticks from the same batch? If no, need to add description on the batch.

**Re:** **All ticks were collected after feeding blood on rabbits, one batch of ticks was used for each experiment, and we add description in the revised MS on tick batch.**

4. The term ‘adult’ is used throughout the paper, including in Fig. 1. But ‘female’ or ‘female adult’ is correct as the strain used is a parthenogenetic strain.
**Re: ‘****adult’ were revised.**

5. Discussion. Lines 348-355 are more appropriately to be included in the Introduction.
**Re: Lines 348-355 were revised.**

6. As results, nymphs were the most sensitive to cinnamaldehyde. A discussion on the reason why, and a comparison of the results with other repellent experiments should be added.

**Re: We did not find literatures to explain this part, and we speculate that it may be due to the more moderate size of the individual nymph. Nymph are more developed than larvae (larvae have three pairs of legs, whereas nymphs and adults have four pairs), and nymphs are more sensitive than adults due to their smaller size and lack of environmental adaptation.**

Minor comments
1. line 84. regents -> reagents

**Re: L84 were corrected.**

2. Line 145. strains -> colonies. Is this correct?

**Re: We have revised** **L145.**

Reviewer #3: Firstly, authors take a result about cinnamaldehyde repellency behavior in tick, they relate this repellency behavior in tick with its Haller's organ, a kind of olfactory sensory structure. Further to analyze a transcriptome data from cinnamaldehyde repellency experiments, they found ionotropic receptor (HL-IR) different expression levels caused the repellency behavior. They characterize this receptor at various. Interfering with HL-IR express level at nymphal stage, they interestingly found that significant decreases in the tick repellency rate from cinnamaldehyde and the response of Haller's organ. To understand the mechanism between HL-IR and cinnamaldehyde repellency behavior, authors with their colleagues validate a interaction effect in above two molecules and explore the critical binding site between the two molecules. Each part of experiment is tightly close. However, certain aspects of the work need to strengthened to ensure all conclusions are well supported.

1) In S1 Fig1C, there is no significant difference between the 0.25% and 0.5% in group of 20min and 40min, but the different labels were signed.
**Re:** **S1 Fig1C were corrected.**

2) In S1 Fig1, please use same type of error bar
**Re: We have revised S1 Fig1.**

3) Couldn't find any structure differences in scanning electron microscope, author reported that larvae lack a sensory hair.

**Re: We replaced the new figure and labeled the sensilla of the anterior pit with an orange arrow in the figure below, with sensilla 6 for larvae and 7 for nymph and adult.**

4) Ionotropic receptor (HL-IR) was a core molecule for further mechanism analysis, and HL-IR was obtained form the transcriptomic research. The research process of how important is this molecule in the repellency behavior effects was weak. In addition to this, the data presentation is very sloppy, greatly detracting for the manuscript. a) Fig note can't correspond to fig tilte in S2 FigA. b) The quantity of transcript of nymph was more than 10 times than other groups in S2 FigA and B, which is unreasonable to analyze intergroup differences. c) 9 transcripts were regarded as "IR-relate" genes in S2 FigC, but this result couldn't be supported with any materials. d) A suggestion about plotting, it could be better if you label "IR-relate" genes on the S2 FigB at the same time.
**Re: a) We modified the S2 FigA tilte. b)Since transcriptome sequencing is intended to screen for particular differential molecules, this section of the paper functions as the supplemental material for this paper. To ensure the validity of transcriptome sequencing, we sent two replicates for each group, and the number of differential transcripts after analysis varied greatly between the groups, which we speculate may be due to the sensitivity to cinnamonaldehyde at different developmental stages. c) After comparing S2 FigC with the genome, we discovered that only five of the nine transcripts were duplicates. We submitted there sequences to Genbank and changed S2 FigC, noting the sequence number in the article's Discussion section.** **d) We attempted to label these genes in S2 FigB, but the way they are shown on the figure makes it difficult to see them.**

5) line 289-291: " To examine its function in the native state,......". I think author just wanted to detected the localization of HL-IR in cell, neither of its function.
**Re: L289-291 were revised.**

6) A interesting phenomenon is that cinnamaldehyde repellency response were decrease after removal of the first pair of legs (Fig1C) but expression of HL-IR were similar among tick's legs (Fig2B). Wouldn't that suggest that HL-IR in Haller's organ were not a major molecule to receive cinnamaldehyde signals?
**Re: We hypothesize that HL-IR is not expressed particularly on the first leg, and that the sensilla on the other legs may express it similarly. However, HL-IR is inscribed on the Haller's organ, which is only found on the first legs.**

7) 2-∆∆Ct method was calculated all RT-PCR experiment data, but I couldn't find in all PR-PCR figure that a given group were normalized to 1. Especially in interferences experiments, the control group should be normalized to 1.

**Re: For RT-PCR experiment data, the Y-axis shows the level of relative expression, and these values are shown directly after the2^-∆∆Ct^ calculation.**

8) Using the competitive fluorescent ligand binding assays to odorant-binding proteins is classic method but limited as tian et al[1] described. Please provide more proof methods.
[1] Tan J, Zaremska V, Lim S, Knoll W, Pelosi P. Probe-dependence of competitive fluorescent ligand binding assays to odorant-binding proteins. Anal Bioanal Chem. 2020 Jan;412(3):547-554.

**Re:** **In insects, the competitive fluorescent binding test is a commonly used method with several drawbacks. To further confirm the binding's validity, we have also shown by molecular docking prediction that they can bind to one another, which was confirmed by targeted mutation. Indeed, proteins with odor molecules were also recorded using a single-cell recording approach, and we attempted to perform whole-cell recordings during cinnamaldehyde stimulation using heterologous expression of HL-IR in HEK 293T cells with reference to prior research studies in order to discover the interaction between HL-IR and cinnamaldehyde . Regretfully, this experiment was unsuccessful. This unsuccessful experiment we mentioned in Discussion.**

9) Does "SSR" referring in line 354 mean single sensillum recording. If so, please provide the full name!
**Re: L354 were corrected.**

10) There are extensive content in second and third paragraphs about Odorant binding proteins, odorant receptors, and gustatory receptor neurons. However, I cannot figure out the correlation between this part and this study. Are the authors trying to demonstrate that HL-IR belongs to one of the three?

**Re:This descriptions were corrected.**
